# Adjuvant and neoadjuvant use of immune checkpoint inhibitors in NSCLC

**DOI:** 10.1007/s00432-023-04749-4

**Published:** 2023-05-13

**Authors:** Anushka Walia, Vinay Prasad

**Affiliations:** 1 University of California San Francisco School of Medicine, San Francisco, USA; 2 Department of Epidemiology and Biostatistics, University of California San Francisco, San Francisco, USA

## Abstract

The neoadjuvant and adjuvant use of immune checkpoint inhibitors (ICIs) in early stage non-small cell lung cancer (NSCLC) is increasing, with pembrolizumab approved as adjuvant therapy following surgical resection and chemotherapy by the U.S. Food and Drug Administration in early 2023. However, clinical trials of these agents have several key limitations including the use of surrogate endpoints that have not been validated and a lack of demonstrated survival benefit. Further data supporting the benefits of ICIs in this setting are necessary to justify their use at the cost of greater financial burdens, time, and adverse effects.

## Editorial

Immune checkpoint inhibitors (ICI) are gaining momentum as adjuvant treatment options for non-small cell lung cancer (NSCLC). On January 26, 2023, the U.S. Food and Drug Administration (FDA) approved pembrolizumab as adjuvant therapy following surgical resection and chemotherapy for stage IB-IIIA NSCLC. This marks the first FDA approval for adjuvant pembrolizumab and the second for adjuvant immunotherapy for NSCLC after atezolizumab’s FDA approval in 2021. This decision was based on results of KEYNOTE-091 (PEARL) that compared pembrolizumab to placebo as adjuvant therapy for completely-resected early stage NSCLC, showing that pembrolizumab was associated with modest improvements in disease-free survival (DFS) (Mauguen et al. 2013). In this commentary, we argue that the set of trials of ICIs in the adjuvant or neoadjuvant space (Table 1), though they show the potential promise of the agents, also highlight limitations of the strategy of broad perioperative application of ICIs.

**Table 1:** Characteristics of key trials of ICIs approved as adjuvant or neoadjuvant therapy in NSCLC

	KEYNOTE-091 (O’Brien et al. 2022)	IMpower010 (Felip et al. 2021)	CheckMate 816 (Forde et al. 2022)
Drug	Pembrolizumab	Atezolizumab	Nivolumab
Setting	Adjuvant	Adjuvant	Neoadjuvant
Approved for	IB—IIIA NSCLC with PD-L1 following chemotherapy	II—IIIA NSCLC with PD-L1 TC ≥ 1% following chemotherapy	Resectable IB—IIIA NSCLC with chemotherapy
Size of ITT population	1177	1005	358
% Patients who received chemotherapy prior to or along with ICI treatment	86%	100%	100%
% Control arm patients receiving subsequent systemic therapy who received ICIs	Not provided	49.6%	64.6%
Primary endpoint(s)	DFS	DFS	EFS, PCR
DFS or EFS HR in ITT population	0.76 (*p* = 0.0014)	0.81 (*p* = 0.040)*	0.63 (*p* = 0.005)
OS HR in ITT population^‡^	0.87 (*p* = 0.17)*	0.995 (*p* = 0.966)*	0.57 (*p* = 0.008)*

*Boundary for statistical significance not crossed

^**‡**^Overall survival data currently immature for all three trials

*PCR* pathological complete response

KEYNOTE-091 is a triple-blind phase 3 trial that randomized patients with stage IB-IIIA NSCLC after complete surgical resection, with or without adjuvant chemotherapy, to receive either pembrolizumab or placebo. Dual primary endpoints were DFS in the overall population and in the PD-L1 tumor proportion score (TPS) of 50% or greater population. In the intention to treat (ITT) population, median DFS was 53.6 months in the adjuvant pembrolizumab arm vs 42.0 months in the placebo arm, with a HR of 0.76. The HR was 0.73 among patients who received adjuvant chemotherapy, and the interaction coefficient was null.

Historically, drug approvals for NSCLC have been granted based on improvements in OS–the gold-standard endpoint in oncology. As median survival in NSCLC increases, surrogate endpoints like DFS have become more popular and are increasingly used as the basis for drug approvals. IMpower010 and CheckMate 816, which led to the FDA approvals of adjuvant atezolizumab and neoadjuvant nivolumab respectively, also used DFS or event-free survival (EFS) as primary endpoints.

Although prior studies have supported DFS as a surrogate for overall survival (OS) in NSCLC, whether these relationships apply to ICIs is unknown. Mauguen et al. examined 60 trials of chemotherapy or radiotherapy in NSCLC, and found strong trial-level correlation coefficients between DFS and OS (*R*^2^ 0.89–0.99). Yet, authors cautioned that “extrapolation to targeted agents is, however, is not automatically warranted” and that “for targeted agents, surrogate endpoints will need to be studied directly in trials of the agents (Mauguen et al. 2013).”

ICIs and TKIs differ from cytotoxic drugs in NSCLC. Cytotoxic drugs cannot cure advanced disease but can increase cure fractions in the adjuvant setting, ostensibly by eradicating microscopic disease. TKIs may merely delay growth and may not lead to cure in either setting–to date, the ADAURA trial evaluating osimertinib for EGFR-positive NSCLC has yet to report OS outcomes. ICIs, in contrast, may lead to durable remissions in both early and advanced disease. These varying properties of therapy classes may lead to vastly different surrogate-survival relationships.

The fact that a surrogate is valid for one category of medications does not mean it is valid for other drug classes. Studies on NSCLC have shown that endpoints like overall response rate (ORR) and progression free survival (PFS) have different associations with OS across trials of targeted therapy, immunotherapy, and chemotherapy (Hua et al. 2022). In addition, there have been recent situations where DFS benefits did not translate to longer OS. In the ADJUVANT trial, gefitinib improved DFS over standard chemotherapy in the adjuvant setting for NSCLC, but 5 year OS rates were not significantly different between treatment groups (Zhong et al. 2021). A meta-analysis of multiple trials on first generation EGFR-mutant NSCLC showed that adjuvant EGFR-TKI therapy improved DFS but not OS compared to chemotherapy or placebo (Chen 2021).

Before DFS is used to justify approvals of ICIs, strong correlations between DFS and clinically-relevant endpoints like OS or health-related quality of life must be established across trials that specifically evaluate ICIs. Demonstration of treatment effects on DFS alone is not sufficient to justify clinical benefit of a drug, especially when DFS improvements were as modest as in the KEYNOTE-091.

In addition, OS data from KEYNOTE-91 is immature. At the time of data collection, 17% of patients in the pembrolizumab arm and 19% of patients in the placebo arm had died. OS rates at 36 months were 82% in the pembrolizumab arm vs. 80% in the placebo arm, and hazard ratios were not statistically significant. Given these early results and the only mild effect of pembrolizumab on DFS, it is not certain that any OS benefit will emerge. Importantly, when OS analysis is presented, it must be carefully evaluated for confounding by post-protocol therapies received. To date, neither atezolizumab nor nivolumab have demonstrated a statistically significant OS benefit in the perioperative setting.

Subgroup analysis of KEYNOTE-091 raises questions about the clinical utility of adjuvant pembrolizumab in key patient populations. The risk of a DFS event in stage IB patients was 21/84 with pembrolizumab vs. 25/85 with placebo. In stage IIIA patients, risk was 89/177 with pembrolizumab and 89/162 with placebo.

Interestingly, DFS hazard ratios favored placebo over pembrolizumab in NSCLC with squamous histology (HR = 1.04, 95% Cl 0.75–1.45) and in patients who did not receive adjuvant chemotherapy (HR = 1.25, 95% Cl 0.76–2.05). DFS improvements also did not depend on PD-L1 TPS, which differs from IMPower010 in which response to atezolizumab was higher in tumors with greater PD-L1 expression.

KEYNOTE-091 results suggest that more than half of patients (327/587) will not experience recurrence even without ICI therapy. These patients experience harms of treatment without possibility of benefit. Consequently, overtreatment is guaranteed with the adjuvant approach. In KEYNOTE-091 pembrolizumab was associated with significant grade 3 or worse adverse events, with adverse events leading to treatment discontinuation in 20% of patients treated. Four participants in the study died due to events attributed to pembrolizumab treatment. The risk–benefit ratio of adjuvant pembrolizumab may not be favorable for a significant portion of patients with NSCLC.

Moreover, in KEYNOTE-091 with extended follow up, DFS curves do not provide evidence of sustained advantage of pembrolizumab in the ITT population. As such, it is unclear if this treatment strategy is increasing cure rate.

ICI therapy is unique in that it is capable of generating durable responses in patients even after recurrence. KEYNOTE-091 has not reported the rates of post-progression ICI use. This is crucial to know, as a key clinical question is whether the same outcomes can be achieved by reserving ICIs for disease progression. In CheckMate 816, the rate of post-protocol ICI treatment in the control arm among those who received systemic therapy was 64.6%, and in IMpower010 it was 49.6%–these percentages should have been 100% to answer this vital question.

The evidence base for adjuvant pembrolizumab, and ICIs in general in the adjuvant and neoadjuvant space, leaves open questions. KEYNOTE-091 demonstrated only mild DFS improvements with adjuvant pembrolizumab and did not provide any OS data, leaving the question of true clinical advantage unanswered. ICI therapy is also associated with significant adverse events and greater risk of treatment discontinuation. Trials of adjuvant atezolizumab and neoadjuvant nivolumab in NSCLC are similarly limited by lack of demonstrated OS benefit and suboptimal post-protocol care. Putting patients through up to a year of extremely costly ICI therapy after surgery and chemotherapy when the advantage may not exist is unethical.

## Data Availability

Data sharing is not applicable to this article as no new data were created or analyzed in this study.
